# Ecological effects of fear: How spatiotemporal heterogeneity in predation risk influences mule deer access to forage in a sky‐island system

**DOI:** 10.1002/ece3.5291

**Published:** 2019-06-02

**Authors:** Christopher Lowrey, Kathleen M. Longshore, David M. Choate, Jyoteshwar R. Nagol, Joseph Sexton, Daniel Thompson

**Affiliations:** ^1^ U.S. Geological Survey Henderson Nevada; ^2^ University of Las Vegas, Nevada Las Vegas Nevada; ^3^ University of Maryland College Park Maryland; ^4^ terraPulse Inc. North Potomac Maryland

**Keywords:** cougar, mule deer, NDVI, predator–prey, RSF, sky islands

## Abstract

Forage availability and predation risk interact to affect habitat use of ungulates across many biomes. Within sky‐island habitats of the Mojave Desert, increased availability of diverse forage and cover may provide ungulates with unique opportunities to extend nutrient uptake and/or to mitigate predation risk. We addressed whether habitat use and foraging patterns of female mule deer (*Odocoileus hemionus*) responded to normalized difference vegetation index (NDVI), NDVI rate of change (green‐up), or the occurrence of cougars (*Puma concolor*). Female mule deer used available green‐up primarily in spring, although growing vegetation was available during other seasons. Mule deer and cougar shared similar habitat all year, and our models indicated cougars had a consistent, negative effect on mule deer access to growing vegetation, particularly in summer when cougar occurrence became concentrated at higher elevations. A seemingly late parturition date coincided with diminishing NDVI during the lactation period. Sky‐island populations, rarely studied, provide the opportunity to determine how mule deer respond to growing foliage along steep elevation and vegetation gradients when trapped with their predators and seasonally limited by aridity. Our findings indicate that fear of predation may restrict access to the forage resources found in sky islands.

## 1 INTRODUCTION

Forage availability and predation risk interact to affect habitat use for a variety of ungulates across biomes (Hamel & Cote, 2007; Lone et al., 2014; Riginos, 2015). Many ungulates must balance the need to acquire sufficient forage required for growth and reproduction while avoiding predation (Kie, 1999). Where predator–prey landscapes consist of distinct hunting grounds and prey refugia (Kaufman et al., 2007), ungulates may shift habitats to trade‐off between predation risk and relative safety (Atwood, Gese, & Kunkel, 2009). Pursuing patchily distributed resources may also result in trade‐offs if some patches are associated with greater predation risk (Lima, 1998), thus affecting ungulate access to quality habitat as well as survival rates (Choate, 2009; Quintana et al., 2016). The influences of resources and risk on habitat selection are expected to change due to seasonal shifts in forage, encounters with predators, and events such as gestation and lactation (Pierce, Bowyer, & Bleich, 2004) with the assumption that animals perceive predation risk as a function of habitat terrain, vegetation type, visibility, and other environmental conditions (Esparza‐Carlos, Laundré, Hernández, & Íñiguez‐Dávalos, 2016; Lima & Steury, 2005; Makin, Chamaillé‐Jammes, & Shrader, 2017).

In the Basin and Range Province of southwestern North America, mountain ranges dominated by conifers are widely separated by desert and shrub steppe regions to form “sky‐island” landscapes, where a suite of species is postulated to have isolated for millennia (Brown, 1971). The relatively greater ecological diversity of sky islands is driven primarily by the elevation gradient that provides increasing precipitation, plant and animal diversity, complex terrain, and refuge from extreme environments (McCormack, Huang, & Knowles, 2009). Although sky islands may serve as refuges of high‐quality habitat during periods of harsh conditions in the surrounding desert, local ungulate populations are contained with their predators and limited by high temperatures during the summer months that coincide with fawning.

Using the normalized difference vegetation index (NDVI) of primary production across different time periods and vegetation types, it is possible to quantify the connections between short‐term increases in NDVI or “green‐up,” animal movements and habitat use, and the timing of reproduction (Hamel, Garel, Festa‐Bianchet, Gaillard, & Coté, 2009; Pettorelli et al., 2011). Species such as mule deer (*Odocoileus hemionus*) are highly dependent on succulent forage to meet energy requirements (Hurley et al., 2014). They are also responsive to NDVI and green‐up in terms of large‐scale movements (Monteith et al., 2014), habitat use (Marshal, Bleich, Krausman, Reed, & Andrew, 2006), and the timing of reproduction (Stoner, Sexton, Nagol, Bernales, & Edwards, 2016). We examined trade‐offs between the intensity of cougar use and use of growing forage within sky‐island habitats of the Mojave Desert. We further examined seasonal changes in mule deer habitat selection with respect to green‐up, changes in the intensity of cougar use, and the timing of reproduction.

Mule deer populations typically pursue higher quality forage during green‐up period(s) (Hebblewhite, Merrill, & McDermid, 2008) and then greatly reduce their foraging and energy expenditure during times of low vegetation growth (Alldredge, Lipscomb, & Whicker, 1974). In the temperate regions of the species’ range, winter is the time of low forage quality, while green‐up occurs during spring and summer (Smith, Krausman, & Painter, 2015). Parturition occurs in early to mid‐summer in many populations (Butler et al., 2009; Smith et al., 2015) and appears to be related to the health of the female before gestation (Bowyer, 1991; Haskell et al., 2008). This gives pregnant mule deer many months to improve nutritional condition both before parturition and during lactation, potentially increasing fawn survival (Lendrum, Anderson, Monteith, Jenks, & Bowyer, 2014; Monteith et al., 2014). In the Mojave Desert, however, green‐up occurs primarily in the winter and spring, and summer is the time of plant desiccation and therefore low forage quality and availability (McKee et al., 2015). This suggests that deer which forage in greening areas earlier in the year would have a selective advantage not seen in more temperate regions. Unless desert mule deer are able to benefit from winter green‐up, the time available to put on weight before parturition and during lactation may be significantly restricted.

Cougars (*Puma concolor*) are a major predator of mule deer within desert ecosystems, and fawns may represent a significant proportion of prey (Logan & Sweanor, 2001). In the isolating circumstances of a sky island, mule deer can neither migrate nor emigrate to safety, essentially making them a resident population under consistent predation risk (Hebblewhite & Merrill, 2009). Mule deer typically reduce risk of predation by early detection and outrunning predators, a strategy that should favor use of open shrub rather than forested habitats available on sky islands (Schmidt & Kuijper, 2015). Females, however, may move to the greater cover of forested areas during parturition to decrease the visibility of fawns and provide thermal protection (Marshal et al., 2006), potentially trading greater forage quality for reduced predation risk and/or heat stress.

In this study, we propose that sky‐island mule deer modify their foraging and fawn‐hiding behaviors to correspond with the timing and availability of plant resources, and that these adjustments can be measured by an analysis of seasonal resource selection functions (RSF). Specifically, we predict female mule deer will demonstrate a pattern of foraging that includes the use of green‐up areas during winter, and that seasonal changes in habitat variable coefficients will reflect a early summer parturition. Additionally, we predict that birth dates of sky‐island mule deer will occur significantly earlier than in populations in more temperate climates, where plant resources are available later in the year. We further propose changes in intensity of cougar use will measurably modify mule deer foraging behavior. Specifically, we predict mule deer will reduce use of growing areas as intensity of cougar use increases especially during summer parturition. In a mule deer RSF model, the trade‐off between green‐up and intensity of cougar use will generate a negative interaction term and the positive relationship between increasing forage quality and female mule deer occurrence will diminish as cougar occurrence increases.

## 2 METHODS

### 2.1 Study area

**Figure 1:**
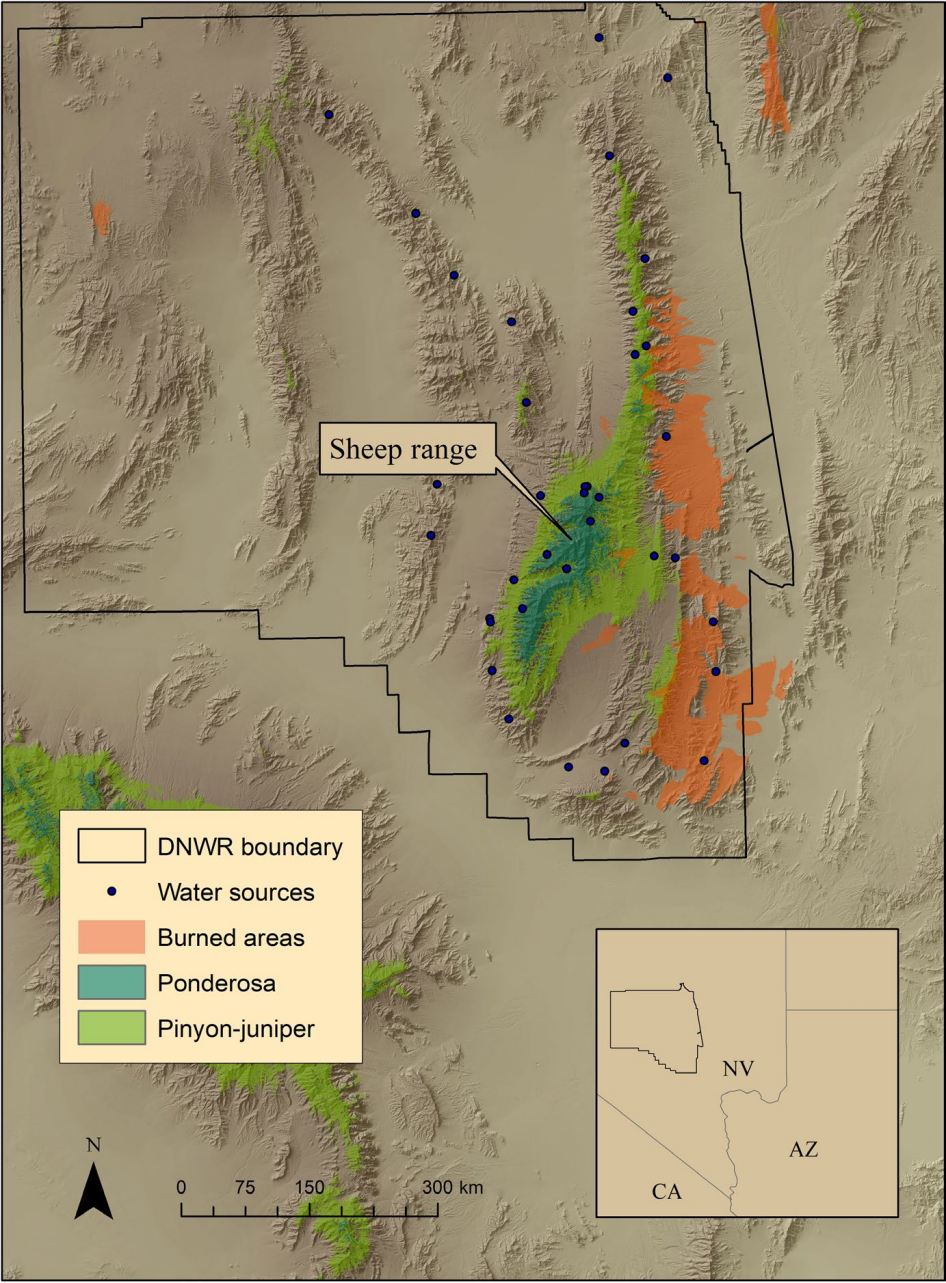
Terrain, vegetation, and burn map and location of Desert National Wildlife Refuge

The Desert National Wildlife Refuge (DNWR) of southern Nevada encompasses 6,540 km^2^ (Figure 1). While the forested, montane environments form the “islands” in a matrix of lowland desert, the complex of desert and ranges creates the sky‐island landscape. Figure 1 depicts the extent of forested areas on the refuge; however, it was beyond the scope of this paper to strictly delineate the boundaries of sky‐island effects. Since both cougar and deer movements extended beyond forested environments, resulting in landscape‐level effects up to the high elevation range, we defined the entire study area as being within the sky island (McCormack, et al., 2009). Precipitation is highly variable (30‐year mean = 11.8 cm, *SD* = 7.8, range: 1.7–37.5) (source: Western Regional Climate Center). Desert shrub is dominant from 800 to 1,800 m and characterized by creosote (*Larrea tridentata*), blackbrush (*Coleogyne ramosissima*), and saltbush (*Atriplex* spp.) associations which often include Mohave yucca (*Yucca schidigera*) and Joshua tree (*Y. brevifolia*). Above 1,800 m, pinyon pine (*Pinus monophylla*) and Utah juniper (*Juniperus osteosperma*) form sparse woodlands which include mountain‐mahogany (*Cercocarpus ledifolius*) and Mormon tea (*Ephedra nevadensis*). Patches of ponderosa pine (*Pinus ponderosa*) occur above 2,200 m. Limestone ridges, cliffs, and rocky outcrops are common. Within the study area, 38 known perennial water sources were available to wildlife including 19 artificial developments and 19 modified natural springs.

### 2.2 Predictor variables

*NDVI*: We used NDVI to estimate vegetation biomass, as an index of forage abundance. NDVI is a satellite‐derived measure of the difference between visible (red) light absorbed by vegetation and the reflectance emitted in the near infra‐red spectrum and is proportional to standing biomass (Pettorelli et al., 2011). Reflectance estimates were derived from the Moderate‐resolution Imaging Spectroradiometer sensor data available from the National Aeronautics and Space Administration. NDVI was averaged within a 500 × 500 m^2^ area (Stoner et al., 2016). *NDVI rate of change (NDVIR)*: We used the rate of change of NDVI between two time periods, two weeks apart as a measure of changes in forage availability (Marshall et al., 2006). *Vegetation type*: A two‐level categorical variable consisting of tree‐ or shrub‐covered areas was derived from the Southwestern Regional Gap Analysis Project. Shrub environments potentially provided more stalking cover for cougars as well as more forage than areas dominated by sparse tree cover. Therefore, we modeled both NDVIR and vegetation type to address the potential relationship between these variables. *Season*: We divided our data set according to three distinct seasonal time periods, based on periods biologically relevant for deer behavior, before analyses. Although there are benefits to running a single model with a season covariate, we believed the clarity of this approach reduced potential confusion in interpreting multiple three‐way interactions necessary in a single‐model approach. February‐May (spring) was the period of greatest vegetation growth; June‐September (summer) included high temperatures, plant desiccation, and greatest reliance on water sources; and October‐January (winter) was the period of greatest precipitation and winter green‐up. We used the interaction terms (cougar rsf [crsf defined below] × NDVI) and (crsf × NDVIR) to measure the effects of our indexes of vegetation biomass and changes in forage availability, respectively, on mule deer's response to cougar intensity of use. *Distance to closest water source*: Water sources were derived from USGS and Nevada Department of Wildlife data and ground‐checked for occurrence of a year‐round water supply. *Distance to closest burned area*: Areas burned, regardless of vegetation type, were delineated using data measured by LandSat sensors from 1987 through 2013 (source: USFS). *Slope percentage*: Slope was measured with a GIS (ArcInfo 10.4) as a ratio of vertical rise/horizontal distance. *Vector ruggedness measure* (VRM): we calculated VRM using a GIS by measuring variation of the three‐dimensional angles within each 10 × 10 m cell covering the study area (Sappington, Longshore, & Thompson, 2007). *Viewshed*: Taken from each animal location, this defines the area within a circle (m^2^) at a given radius minus that area obscured from view by topography. *Profile curvature*: The shape of the slope is in the direction of the maximum slope. Negative values are upwardly convex, and positive values are upwardly concave. *Planform curvature*: The shape of the slope is in the direction perpendicular to the maximum slope. Positive values are laterally convex, and negative values are laterally concave. *Principal component analysis *(PCA) *to reduce multicollinearity of terrain variables*: We used the first principal component (PC1) as a latent variable for the four highly correlated terrain variables of slope, VRM, viewshed, and elevation (Table 1). PC1 accounted for 75.8% of the variation in a PCA of these four variables with positive PC1 values representing the common covariance of increasing slope, ruggedness (VRM), and elevation and decreasing viewshed. Curvature variables were uncorrelated with the other terrain variables and therefore were not included in the PCA. PC1 represents the expected correlations between topographic variables which arise due to geomorphological processes that generate relatively scale‐independent relationships between slope, valleys, and drainage areas (Montgomery & Dietrich, 1992). Increases in elevation from the base of a mountain are typically associated with increased slope and increased erosion of hills, gullies, and valleys which culminate near the tops of drainages at all scales (Istanbulluoglu, Yetemen, Vivoni, Gutiérrez‐Jurado, & Bras, 2008) and cause increased vector measured ruggedness. Ruggedness is necessarily negatively related to viewshed due to topographic obstruction of view. To decouple the intercorrelated terrain variables but retain the ability to interpret different components of topography, we borrow a method from morphometrics and allometry (Bookstein, 1989) and remove the effects of the covarying variables by generating measures of relative terrain shape in their respective units with the residuals from regressions of each terrain variable against PC1. *Slope, VRM, and Viewshed Residuals*: Having accounted for 75.8% of the variance with PC1, we quantified residual slope, VRM, and viewshed relative to the common covariance of PC1 with separate linear regressions of each variable against PC1. These residual values represent the relative slope, VRM, or viewshed, which exceeds, either positively or negatively, that which is accounted for by PC1. Including PC1 and all four residual terrain variables in the linear model would create a perfect representation error. Therefore, residual elevation was excluded based on its relatively low PC loading and the difficulty of interpreting deviations in elevation.

**Table 1:** Pearson correlation values between slope, elevation, VRM, and viewshed variables within the Desert National Wildlife Refuge, Nevada

	Viewshed	Slope	Elevation	VRM
Viewshed				
Pearson Correlation	1	−0.647	−0.389	−0.573
Sig. (2‐tailed)		<0.001	<0.001	<0.001
Slope				
Pearson Correlation	−0.647	1	0.475	0.816
Sig. (2‐tailed)	<0.001		<0.001	<0.001
Elevation				
Pearson Correlation	−0.389	0.475	1	0.356
Sig. (2‐tailed)	<0.001	<0.001		<0.001
VRM				
Pearson Correlation	−0.573	0.816	0.356	1
Sig. (2‐tailed)	<0.001	<0.001	<0.001	

Note

Values were derived from 46,000 random locations.

Abbreviation: VRM, Vector ruggedness measure.

*Pooling data across years*: Average monthly climatological values within the DNWR were not significantly different between years or between respective seasons (i.e., spring of one year vs. spring of another) for precipitation (*F*_1,23_ = 0.51, *p* = 0.48; mean yearly = 11.7 cm, *SD* = 1.5), average daily temperature (*F*_1,23_ = 0.04, *p* = 0.85; mean = 18.6 C, *SD* = 8.8), or maximum temperature (*F*_1,23_ < 0.01, *p* = 0.99; mean = 30.6 C, *SD* = 9.1). We therefore pooled our data across the study period. To compare the contribution of each variable to mule deer occurrence, all variables were standardized (mean—observation/standard deviation) across the entire dataset before separation into seasons (McGarigal, Cushman, & Stafford, 2000).

### 2.3 Mule deer captures

Capture techniques for all animals were performed under guidelines for the use of live animals (Sikes & Gannan, 2011) and followed protocols approved by the University of Nevada, Las Vegas’ Institutional Animal Care and Use Committee. We captured 19 adult (>3 years old) female mule deer during December 2012 and January of 2013. Mule deer were netted from a helicopter and hobbled to prevent injury. No drugs were used. Animals were aged by tooth wear, weighed, fitted with GPS‐satellite collars (Telonics Gen4) and then released on site within 15 min of capture. Collars recorded locations once every four hours starting at 12 a.m. for 30 months and were equipped with an automatic release and mortality sensor.

### 2.4 Cougar captures

Cougar captures occurred from October 2010 through May 2012. We spread trapping efforts across the study area and supplemented trapping with hound/tracking surveys for the more remote regions. We employed any of the following three methods depending on animal safety (e.g., ambient temperatures, terrain) and logistical considerations: Hounds pursued the cougar until it sought refuge in a tree or cliff (Hemker, Lindzey, & Ackerman, 1984); foot‐hold snares were placed at kill sites or along cougar travel routes (Logan, Sweanor, Smith, & Hornocker, 1999); or, when access enabled transport, we set cage traps baited with deer carcasses (Bauer, Logan, Sweanor, & Boyce, 2005). Cougars were immobilized with a combination of 2 mg/kg ketamine and 0.2 mg/kg medetomidine (Kreeger, Raath, & Arnemo, 2002) and held for less than one‐half hour while being fitted with a GPS‐satellite collar (Telonics Gen4). Collars were placed on adult (>3 years) or subadults (1.5–3 years old) only. Collars acquired a location once every four hours starting at 12 a.m. for 24 months and were equipped with an automatic release mechanism and mortality sensor.

### 2.5 Cougar intensity of habitat use or cougar RSF

We randomly selected 6,050 locations from a total of 7,672 collected over the two‐year period and used a 1:1 ratio of used versus available points in a binary logistic model to determine the cougar resource selection function (RSF) or intensity of use within each season. A priori candidate models (for both cougar and deer, below) were derived based on ecological assumptions of terrain structure (hiding/escape), use of water, vegetation type (structure/forage), burned areas (edge effects/forage), and NDVI (forage). AIC model selection was used to rank seven candidate models in SPSS (IBM corp.; Burnham & Anderson, 2002). The highest‐ranked model was used to calculate the cougar RSF values, which were used as an estimate of cougar predation risk. Model performance was measured with overall likelihood ratio χ^2^, goodness of fit values (deviance, Pearson χ^2^), and the area under the curve (AUC) of the receiver operating characteristic (ROC; Hanley & McNeil, 1982).

Home ranges were derived for each cougar each season using a 95% Gaussian kernel density estimator and smooth cross‐validation to estimate bandwidth. We combined individual cougar home ranges by season to determine the perimeter outlines of the seasonal home ranges and used these outlines to constrain random points. Although bighorn sheep (*Ovis canadensis nelsoni*) are a commonly exploited alternative prey species in this system, our preliminary analysis indicated bighorn occurrence did not contribute strongly enough for inclusion as a variable in the candidate models.

### 2.6 Mule deer habitat selection

We randomly selected 44,240 mule deer locations from the 91,303 collected over the study period and used a 1:1 ratio of used versus available points in a binary logistic model to determine the mule deer RSF. Given that our hypotheses dealt with the interaction of available forage and predation risk, we chose the cumulative home range of the collared cougar population as the most appropriate scale of analyses of habitat use to prevent exclusion of areas available to mule deer. In addition to the predictor variables and treatment of correlated variables described previously, we used three interaction terms: *vegetation type* × *cougar RSF*, *season* × *cougar RSF*, and a *cougar RSF* × *change in NDVI* term to determine whether mule deer are trading forage quality for predation risk. Out of six candidates, we used the highest AIC‐ranked model to calculate mule deer RSF values.

### 2.7 Estimation of fawn birth dates

We placed cameras (Bushnell) at 35 water sources throughout the study area. Of these, 10 consistently photographed mule deer. From these 10, we selected two cameras within each of the four following habitat types along an elevation gradient: desert shrub, Joshua tree, pinyon‐juniper, and ponderosa pine associations. Fawn birth dates were estimated from appearance of spotting in coats and nursing behavior (Anderson & Wallmo, 1984).

## 3 RESULTS

### 3.1 Biomass and green‐up

**Figure 2:**
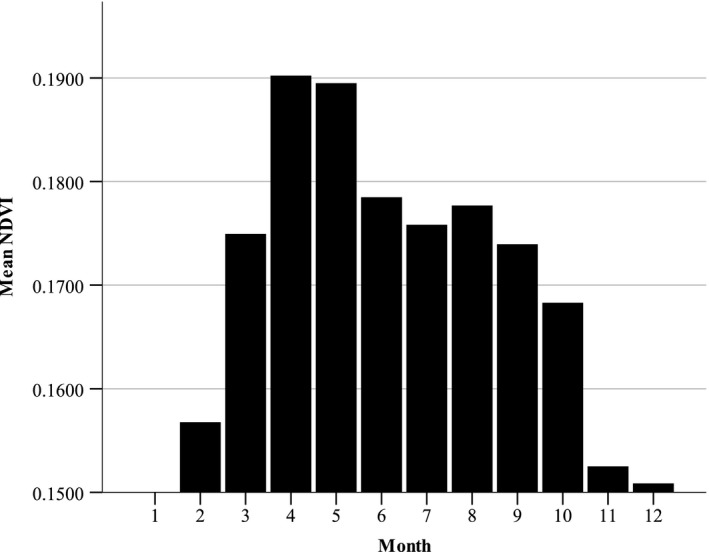
Mean monthly normalized difference vegetation index derived from random points within mule deer home ranges, Desert National Wildlife Refuge, Nevada

**Figure 3:**
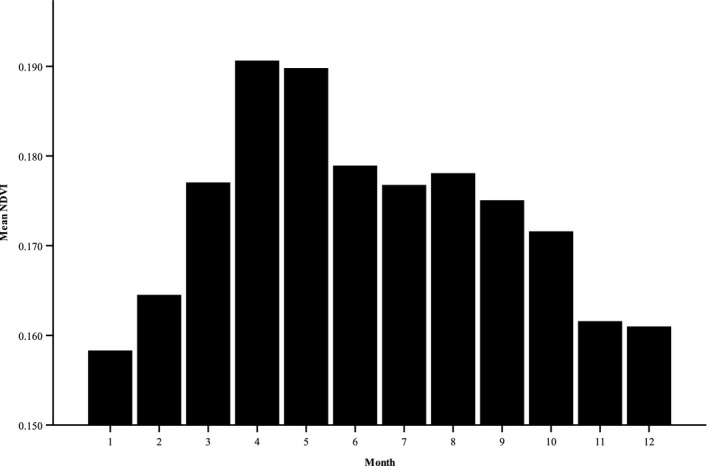
Mean two‐week change in normalized difference vegetation index derived from random points within mule deer home ranges, Desert National Wildlife Refuge, Nevada. Positive values indicate vegetation growth, whereas negative values indicate desiccation

Mean biomass (NDVI) had the lowest value in January, increased to a peak in April and May, then declined continuously to a low in the winter months (Figure 2). The mean two‐week change in NDVI or green‐up (NDVIR) was greatest from late winter to early spring with a peak in March. Subsequently, mean NDVIR declined and became negative with plants desiccating in May and June, increased again with modest green‐up in late summer, then declined in October. The green‐up during most summer months, though positive, was relatively low (Figure 3).

### 3.2 Cougar RSF or intensity of use model selection

**Figure 4:**
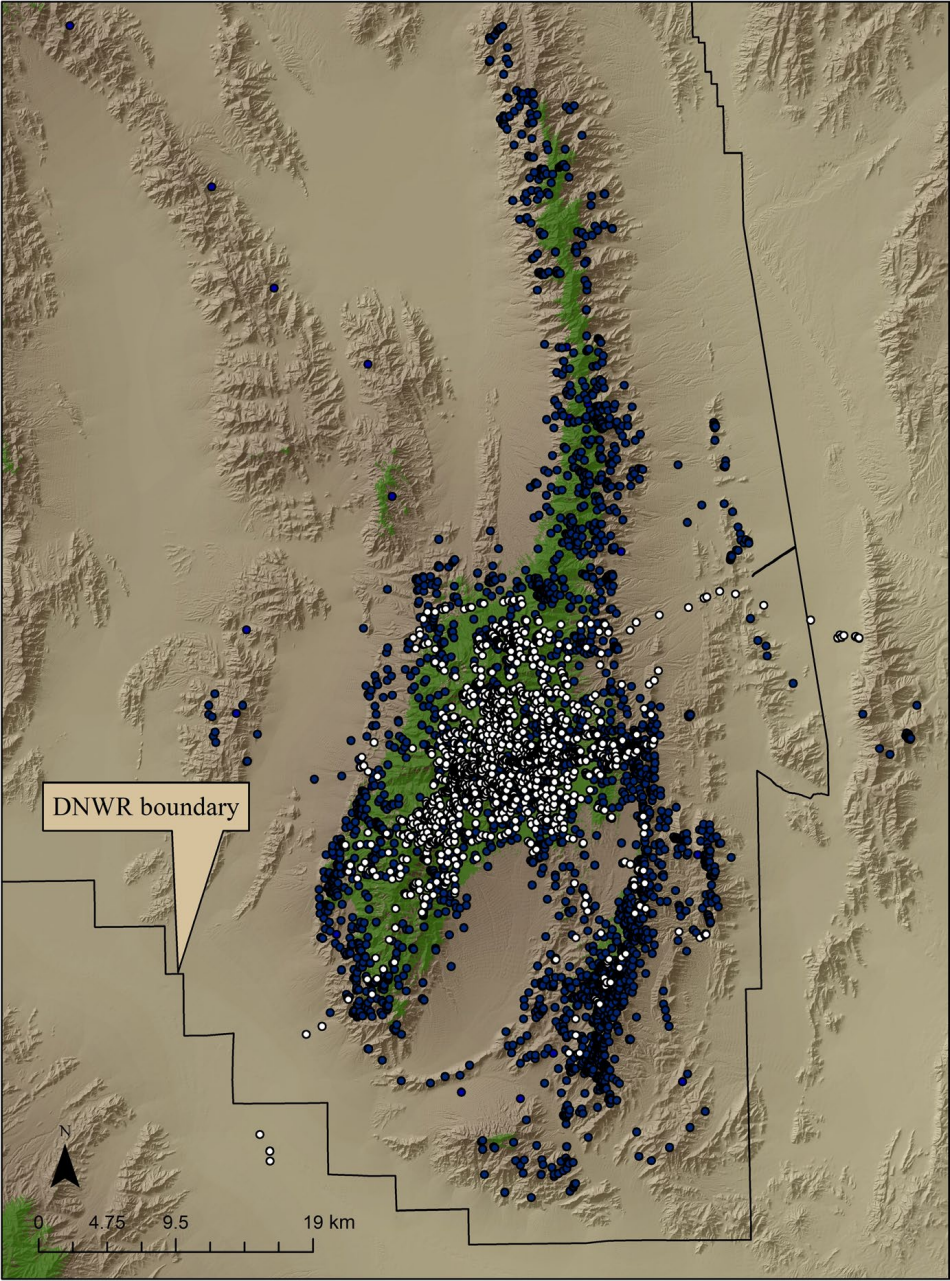
Cougar locations (2010–2012) on the Desert National Wildlife Refuge, Nevada. Dark locations are from winter (October‐January) and spring (February‐May), white locations are from summer (June‐Sept). Green indicates forested areas

We captured 4 female cougars (2 adults, 2 subadults) and 1 subadult male and equipped each with a GPS radio‐collar. During winter and spring, cougars were widely distributed across the region, primarily in the Sheep Range. However, we observed a concentration of use on the central forested areas during summer (Figure 4). During spring and summer, the model containing all measured variables except curvature planform best explained cougar habitat use (spring AUC = 0.882, 95% CI = 0.870–0.893, likelihood χ^2^ = 2,472.5, *p* < 0.001. Deviance = 0.911, Pearson χ^2^ = 1.085). Summer AUC = 0.958, 95% CI = 0.952–0.965, likelihood χ^2^ = 3,398.9, *p* < 0.001. Deviance = 0.804, Pearson χ^2^ = 2.072; Table 2). Within the winter season, we found two models contained reasonable evidence (delta AIC < 2.0) for explaining habitat selection. The first contained all variables except curvature planform, and the second contained all variables except NDVI. We chose the best model based on performance (AUC = 0.881, 95% CI = 0.869–0.893, likelihood χ^2^ = 2,254.5, *p* < 0.001. Deviance = 0.818, Pearson χ^2^ = 1.12). Although averaging across reasonable models when using AIC is an option, it would preclude our ability to critically evaluate the relative contribution of individual coefficients from logistic regression (Cade, 2015). We used the highest‐rated models to estimate cougar RSF values (cougar intensity of use) within each respective season.

**Table 2:** Cougar seasonal habitat use candidate models

Cougar candidate models	Spring Delta AIC	Summer Delta AIC	Winter Delta AIC
PC^a^ + dwater^b^ + veg^c^ + sloperes^d^ + vrmres^e^ + viewres^f^ + curvpro^g^+ndvi^h^ + dburn^i^	0.0	0.0	0.0
PC + dwater + veg + sloperes + vrmres + view res + curvpro + curvpl^j^ + ndvi + dburn	3.4	2.5	2.3
PC + dwater + veg + sloperes + vrmres + viewres + curvpro + curvpl + ndvi	358.0	54.9	322.2
PC + dwater + veg + sloperes + vrmres + viewres + curvpl + ndvi + dburn	7.0	46.1	20.5
PC + dwater + veg + sloperes + vrmres + viewres + curvpro + curvpl + dburn	33.6	37.8	1.3
PC + dwater + sloperes + vrmres + viewres + curvpro + curvpl + ndvi + dburn	4.2	5.2	2.1
PC + veg + sloperes + vrmres + viewres + curvpro + curvpl + ndvi + dburn	7.9	20.8	48.8

Notes

Desert National Wildlife Refuge, Nevada. The first model in the list was the highest ranked for all seasons.

Spring = February–May, summer = June–September, winter = October–January.

a PC = principal component 1 analysis score of slope, ruggedness (VRM), viewshed, and elevation.

b dwater = distance to water sources.

c veg = vegetation types (trees or shrubs).

d sloperes = slope residual.

e vrmres = ruggedness residual.

f viewres = viewshed residual.

g curvpro = curvature profile.

h ndvi = normalized difference vegetation index.

i dburn = distance to previously burned areas.

j curvpl = curvature planform.

### 3.3 Mule deer model selection

During spring, AIC selection derived two models with reasonable evidence for explaining mule deer habitat use. The strongest model included all predictors except distance to previously burned areas. The second reasonable model indicated that all measured variables contributed to habitat use. We chose the strongest model for this and the two subsequent seasons based on performance (likelihood ratio χ^2^ = 4,307.4, *p* < 0.001. AUC = 0.718, 95% CI = 0.713–0.724. Deviance = 1.252, Pearson χ^2^ = 0.991).

During summer, two models had reasonable evidence. The strongest model included all predictors except the cougar RSF × NDVI interaction term (likelihood ratio χ^2^ = 15,704.5, *p* < 0.001. AUC = 0.913, 95% CI = 0.909–0.0916. Deviance = 0.877, Pearson χ^2^ = 1.672). The second‐highest rated included all variables (Table 3).

**Table 3:** Female mule deer seasonal habitat use candidate models and AIC Delta values

Mule deer candidate models	Spring Delta AIC	Summer Delta AIC	Winter Delta AIC
PC^a^ + dwtr^b^ + ndvi^c^ + ndvir^d^ + veg^e^ + slpres^f^ + vrmres^g^ + vwres^h^ + crsf^i^ + dstburn^j^ + (crsf × ndvir)^k^ + (crsf × ndvi) + (veg × crsf) + (veg × ndvir)	322.4	69.6	0.0
PC + dwtr + ndvi + ndvir + veg + slpres + vrmres + vwres + crvpro^l^ + crvpl^m^ + crsf + (crsf × ndvir) + (crsf × ndvi) + (veg × crsf) + (veg × ndvir)	0.0	447.6	452
PC + dwtr + ndvi + ndvir + veg + slpres + vrmres + vwres + crvpro + crvpl + crsf + dstburn + (crsf × ndvi) + (veg × crsf) + (veg × ndvir)	520	184.4	161.8
PC + dwtr + ndvi + ndvir + veg + slpres + vrmres + vwres + crvpro + crvpl + crsf + dstburn + (crsf × ndvir) + (crsf × ndvi) + (veg × crsf) + (veg × ndvir)	1.9	0.7	2.1
PC + dwtr + ndvi + ndvir + veg + slpres + vrmres + vwres + crvpro + crvpl + crsf + dstburn + (crsf × ndvir) + (crsf × ndvi) + (veg × ndvir)	47.1	9.8	79.1
PC + dwtr + ndvi + ndvir + veg + slpres + vrmres + vwres + crvpro + crvpl + crsf + dstburn + (crsf × ndvir) + (veg × crsf) + (veg × ndvir)	99.3	0.0	3.7
PC + dwtr + ndvi + veg + slpres + vrmres + vwres + crvpro + crvpl + crsf + dstburn + (crsf × ndvi) + (veg × crsf)	1,334.3	3,937.4	1,932.9
PC + dwtr + ndvir + veg + slpres + vrmres + vwres + crvpro + crvpl + crsf + dstburn + (crsf × ndvir) + (veg × crsf) + (veg × ndvir)	200.4	2080.7	1,154.5

Note

Desert National Wildlife Refuge, Nevada.

Spring = February‐May, summer = June‐September, winter = October‐January.

a PC = principal component 1 analysis score of slope, ruggedness (vrm), viewshed, and elevation.

b dwater = distance to water sources.

c ndvi = normalized difference vegetation index.

d ndvir = rate of change in ndvi over a two‐week period.

e veg = vegetation types defined as trees (reference category) or shrubs.

f slope res = slope residual.

g vrmres = ruggedness residual.

h viewres = viewshed residual.

i crsf = cougar relative probability of occurrence.

j dburn = distance to burned areas.

k x indicates interaction between terms.

l curvpro = curvature profile.

m curvpl = curvature planform.

During winter two models, one containing all variables except curvature profile and planform and the other containing all variables had reasonable evidence for explaining habitat selection (likelihood ratio χ^2^ = 6,270.4, *p* < 0.001. AUC: 0.809, 95% CI = 0.803–0.814. Deviance = 1.083, Pearson χ^2^ = 1.066).

### 3.4 Mule deer habitat selection

**Figure 5:**
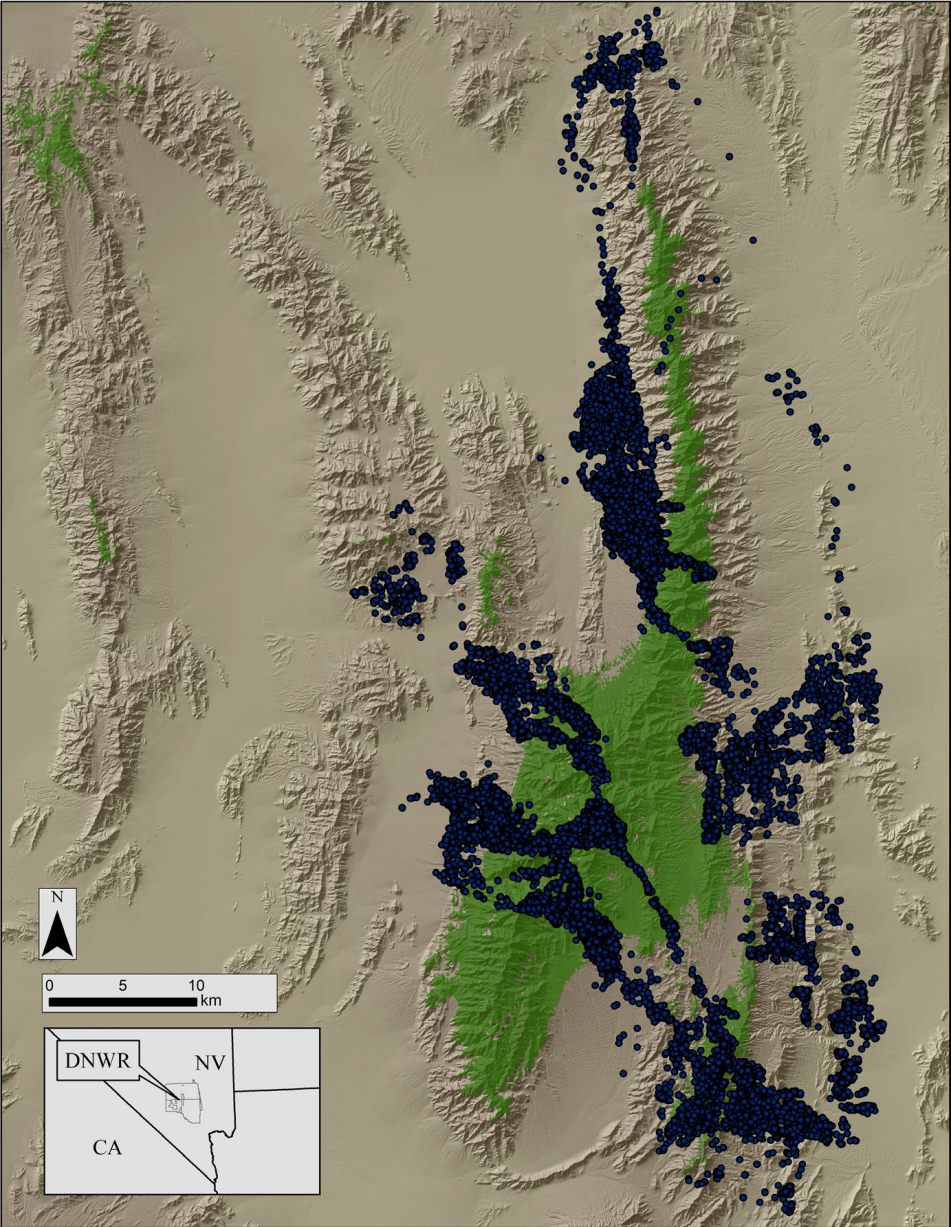
Female mule deer locations (2010–2012) on the Desert National Wildlife Refuge, Nevada. Green indicates forested areas

Female mule deer used a wide variety of terrain within the study area (Figure 5). During spring, mule deer responded positively though weakly to biomass (NDVI), and positively to green‐up (NDVIR). Mule deer were associated with an increasing likelihood of cougar occurrence, suggesting similar habitat use (Table 3). Interestingly, cougar intensity of use interacted strongly and negatively with NDVIR, indicating that as cougar use increased, the positive effect of NDVIR on mule deer occurrence diminished. PC scores and residual effects indicated deer used relatively lower slopes in less rugged areas of greater viewshed. Curvature profile was positive and planform negative, indicating greater use of valley and ravine areas versus ridgelines. Mule deer were associated with water though not as strongly as we found in other seasons. Shrubs were used more commonly than tree‐covered habitats (81% of deer locations), although distance to previously burned areas did not contribute to habitat selection. Mule deer appeared more strongly influenced by predation risk within shrub than tree‐covered areas during spring (Table 4).

**Table 4:** Female mule deer seasonal habitat model variable coefficient (beta) and odds ratios within the Desert National Wildlife Refuge, Nevada

Variables	February–May			June–September			October–January		
	Beta^a^	*SE* b	Odds ratios	Beta	*SE*	Odds ratios	Beta	*SE*	Odds ratios
PCsrve^c^	−0.479	0.02	0.619	−0.590	0.03	0.554	−0.578	0.05	0.561
Dwater^d^	−0.112	0.01	0.894	−0.834	0.02	0.434	−0.189	0.02	0.828
Dburn^e^	N/A	N/A	N/A	−0.475	0.02	0.622	−0.403	0.02	0.669
Veg^f^	0.431	0.09	1.423	−0.216	0.12	0.805	0.326	0.08	1.386
Slope resid^g^	−0.319	0.02	0.727	−0.114	0.02	0.892	−0.176	0.02	0.839
VRM resid^h^	−0.400	0.02	0.671	−0.131	0.03	0.877	−0.135	0.02	0.874
Viewshed resid^i^	0.080	0.01	1.086	0.292	0.02	1.340	0.260	0.02	1.297
Curv profile	0.223	0.02	1.250	0.157	0.02	1.157	N/A	N/A	N/A
Curv planform	−0.123	0.02	0.884	−0.083	0.02	0.920	N/A	N/A	N/A
NDVI^j^	0.154	0.04	1.166	1.957	0.06	7.078	1.249	0.04	3.486
NDVIR^k^	0.223	0.02	1.262	−0.339	0.02	0.712	−0.374	0.02	0.688
CRSF^l^	0.285	0.30	1.329	0.261	0.03	1.299	0.433	0.03	1.542
NDVI × CRSF^m^	0.212	0.02	1.236	N/A	N/A	N/A	−0.044	0.02	0.957
NDVIR × CRSF	−0.390	0.01	0.677	−0.216	0.01	0.805	−0.201	0.01	0.818
CRSF × Veg^f^	−0.395	0.11	0.673	−0.186	0.07	0.830	−0.483	0.05	0.617
NDVIR × Veg^f^	−0.252	0.05	0.778	−0.836	0.05	0.433	−0.608	0.05	0.545

a Beta coefficient, standardized by the standard deviation.

b *SE* = standard error.

c Principal component 1 of slope, ruggedness, viewshed (loading opposite in sign), and elevation.

d Dwater = distance to water in meters.

e Dburn = distance to burned areas.

f Veg = categorical shrub relative to tree (reference) vegetation types.

g Residual from regression between slope and principal component 1 scores.

h residual from regression between VRM and principal component 1 scores.

i Residual from regression between viewshed and principal component 1 scores.

j NDVI = normalized difference vegetation index.

k NDVIR = rate of change in NDVI between two samples taken two weeks apart.

l CRSF = cougar resource selection function.

m × Denotes interaction between adjoining variables.

During summer, mule deer were strongly and positively associated with NDVI, a result coinciding with the increased use of forested areas. However, the effect of NDVIR was negative, likely indicating the reduced availability of green‐up in the summer months. A negative interaction between cougar intensity of use and NDVIR continued through the summer, suggesting greater risk of predation for mule deer within any remaining growing areas. Similar to spring, summer PC scores and residual effects indicated deer used relatively lower slopes in less rugged areas of greater viewshed. Curvature values again indicated greater use of valley and ravine areas versus ridgelines. Mule deer were strongly associated with previously burned areas in the summer months, and distance to permanent water sources decreased as expected. Mule deer shifted from using primarily shrub‐covered areas to using both tree (53% of total use) and shrub areas (46%). Cougar habitat use followed this shift, with an almost equal use across both tree and shrub habitat types (Table 4).

During winter, mule deer maintained a positive association with relatively greater biomass, generally shifting habitat use toward primarily shrub‐covered areas (69% of deer locations). The effect of NDVIR was again negative, suggesting mule deer consistently follow vegetation green‐up primarily in spring. However, this finding may be affected by the scale at which NDVIR was measured. Negative interaction between cougar use and NDVIR continued during winter, suggesting a consistent relationship between cougar intensity of use and areas of growing vegetation. Winter PC scores and residual effects indicated deer used relatively lower slopes in less rugged areas of greater viewshed, though this relationship was not as strong as in other seasons. Curvature did not contribute to the winter model, indicating no detectable difference in use of valley areas versus ridgelines. As in summer, mule deer were positively associated with burned areas, suggesting that these areas are important for most of the year. Distance to water was similar to springtime occurrence. Cougar habitat use again followed the shift toward shrub‐covered areas, with risk greater in the shrub relative to tree‐covered regions (Table 4).

### 3.5 Estimation of fawn birth dates

We viewed over 28,700 photos over 36 months incorporating the study period (including 3 months before and after). From the estimated ages of fawns viewed, we determined birth dates within the relatively lower elevation desert shrub and Joshua tree associations to be from late May to early June, and within the higher elevation pinyon‐juniper and ponderosa pine associations to be from early June to early July.

## 4 DISCUSSION

Based on mule deer association with NDVI and NDVI rate of change, we found little support for the hypothesis that female mule deer follow available green‐up during the winter months, but instead found deer used this strategy primarily during the spring. In this sky‐island population, a narrow four‐month period stands in contrast to other western populations of mule deer, for which foraging on growing plants continues through the summer (Bowyer, 1991). The restricted availability of quality forage for lactating females may negatively impact nutritional requirements (Parker, Gillingham, Hanley, & Robbins, 1999), fawn survival (Monteith et al., 2014; Parker, Barboza, & Gillingham, 2009), and therefore population dynamics (Cook et al., 2004; Garrott, White, Bartmann, Carpenter, & Alldredge, 1987). A small but perhaps important increase in available NDVIR corresponded with an increase in use of burned areas, suggesting deer attempted to prolong access to growing forage into the summer months (Peek, Riggs, & Lauer, 1979). Greater use of burned areas continued into the winter months, further supporting the inference that these areas provide greater forage availability (McCullough, 1969).

Southwestern forest interiors generally provide lower forage‐plant density than that available in shrub‐covered areas (Altendorf, Laundre, Gonzales, & Brown, 2001). As such, the summer shift from deer using primarily shrubs to both shrub and forested areas coincided with a declining use of available growing vegetation. The shift toward use of habitat with higher tree cover potentially provided shade and thermal buffering as well as cover to avoid detection of fawns during parturition (Monteith et al., 2014). This habitat shift, along with the diminished availability of growing vegetation, effectively began a period of low‐quality forage use in summer months. Our data indicated the timing of parturition occurred primarily in early to mid‐June. This was similar to the timing in other populations for which the availability of growing forage generally occurs during spring and summer (Bowyer, 1991; Monteith et al., 2014). This implies Mojave Desert mule deer have not synchronized parturition to the relatively earlier growing season found on sky islands, resulting in a shortened period of forage availability during lactation (Bowyer, 1991). Alternatively, lactating females may have been able to take advantage of the two‐month green‐up occurring after summer rains. However, our data, which we analyzed over 4‐month periods, detected no such relationship. Distance to water sources greatly declined in summer, and the need for water may also explain a shift in habitat use (Longshore, Lowrey, & Thompson, 2009; Ordway & Krausman, 1986). However, because water is widely distributed throughout the Sheep Range, with 28 of 38 permanent sources occurring within shrub habitat, water use does not likely explain the shift toward forested areas.

Although we recognize our data are correlative, we found consistent support for our hypothesis that cougar intensity of use influenced both mule deer foraging and habitat use. There was a broad overlap between cougar and mule deer habitat use during spring, as both species used a sympatric range of terrain, elevation, and vegetation types (Hebblewhite & Merrill, 2009). We found the negative effects of cougar occurrence on the ability of mule deer to use greening areas were greatest during spring. However, as indicated by a positive association between mule deer occurrence and NDVIR, deer nonetheless benefitted from spring vegetation growth. Mule deer may be willing to trade the risk of cougar predation for the seasonally greater benefit of growing vegetation occurring in spring (Festa‐Bianchet, 1988). This inference is intuitive, as the greater availability and wider distribution of growing areas in the spring months necessarily reduced the relative patchiness of resources, and therefore risk, to foraging animals.

**Figure 6:**
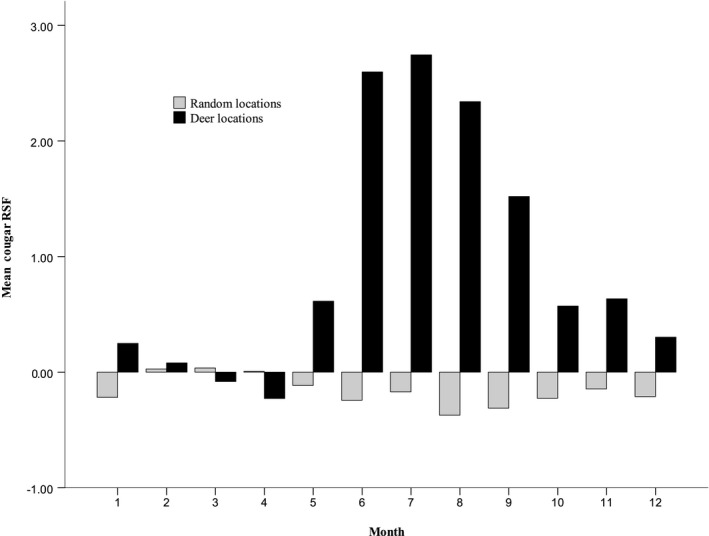
Resource selection function values for cougars at mule deer and random locations within the Desert National Wildlife Refuge, Nevada. RSF values are not scaled between 0–1 and so contain negative values

Cougar intensity of use, although still slightly greater in shrub‐covered areas, increased in forested areas during the summer as cougars presumably focused predation activities near fawning sites (Pierce, Bleich, & Bowyer, 2000) and/or increased elevation to reduce heat load (Figure 6). The seasonal concentration of both mule deer and cougar locations within the forested habitats in summer and the resulting increase in cougar occurrence suggest female mule deer were unable to avoid cougars by shifts in habitat use (Laundre, 2010). However, it is unknown whether fawns born in forests or shrub‐covered areas were more vulnerable to predation in the sky island. Evidence from our analysis, although correlative, suggests that an increase in cougar occurrence detrimentally affected the ability of mule deer to use greening vegetation in summer. This finding, combined with negative mean NDVIR in summer, indicates that when greening vegetation is, on average, low in availability or declining, deer are less likely to risk predation to procure it (Hebblewhite & Merrill, 2009).

In winter, female mule deer shifted away from forest toward shrub‐covered habitats and increased their use of previously burned areas. Interestingly, although the availability of growing areas increased beginning in November, mule deer use of these areas remained relatively low. Cougar intensity of use, measured by the cougar RSF, declined at mule deer locations during winter. Mule deer, however, reduced their response to areas that had measurable green‐up (negative NDVIR × CRSF interaction) during winter. This supports the hypothesis that mule deer are less likely to trade‐off predation risk for greater forage availability during both the summer and winter (Hebblewhite & Merrill, 2009). Reduced use of growing areas (negative NDVIR) and increasing use of areas of greater vegetation coverage (strongly positive NDVI) indicate mule deer were more reliant on browse than growing vegetation during winter (Bowyer, 1991). These findings reinforce our interpretation that although growing vegetation was available during winter, perceived predation risk precluded consistent use of these resources by deer (Barnier et al., 2014; Creel, Winnie, Maxwell, Hamlin, & Creel, 2005). Overall, our results suggest sky‐island mule deer have not developed a strategy for maximizing nutritional resources while minimizing risks. Access to digestible forage, measured here as growing vegetation, has been shown to be an important limiting factor in female mule deer nutrition (Parker et al., 1999). As a result, vital nutritional reserves for pregnant females may be suppressed by an increase in cougar occurrence, potentially resulting in reduced reproductive success in Mojave sky‐island landscapes occupied by cougars (Parker et al., 2009).

## CONFLICT OF INTEREST

None declared.

## AUTHOR CONTRIBUTIONS

CL, KL, DC, JS, and DT conceived the ideas and designed the methodology. CL, KL, and DC collected the data. CL, JN, and DT analyzed the data. CL, KL, DC, and DT led the writing of the manuscript. All authors contributed critically to the drafts and gave final approval for publication.

## Data Availability

All data used for this manuscript will be available to the public within the U.S. Geological Survey's Science Base data archive (https://www.sciencebase.gov; https://doi.org/10.5066/P9CC5E8P; Lowrey, Longshore, Choate, Sexton, & Nagol, 2019).
